# Medical Advertising

**Published:** 1909-04-03

**Authors:** 


### Medical Advertising.

Some years ago a well-known London Club, which exists, if we are correctly informed, for the purpose of furthering the cause of judicious and legitimate advertising, invited a number of prominent members of the medical and legal professions to participate in a discussion on the desirability of eliminating the existing conventional clauses that regulate the conduct of members in these professions in regard to the advertisement columns. The symposium that resulted from this invitation proved most interesting, and it is a pity that no full shorthand note of the speeches is extant. There was, on the part of Club members—all of them " common-sense, practical, commercial-minded men "—a natural surprise and astonishment at the attitude which lawyers and doctors are compelled to adopt, and an equally natural scepticism as to the good results which spring from existing regulations. Nor, to a layman at least, could the defence of the anti-advertising professionals be regarded as adequate and fair. It is reasonable to suppose that members of the profession, many of whom are, after all, also " practical, common-sense, commercial-minded men "—and we use the last qualifying adjective in its highest and most commendable sense—are not indifferent to the arguments which were brought forward at that symposium, and that many a medical man has at times troubled his brain and vexed his conscience by secretly deploring the conventions that prevent frank advertisement. It is, perhaps, time that the subject should be reconsidered. It is at least time that some authoritative pronouncement should be made on what constitutes proper advertisement, and what not. We are quite aware that various bodies have laid upon themselves the task of posing as the mentors of their colleagues in this matter. An American association has formulated a lively ethical code to regulate the conduct of its members. Yet at the present moment there are members of the profession in the States whose names appear daily in half a column of print under headlines which, to a sober-minded Englishman, savour of the acme of fulsome appreciation. With us, also, ethical codes have been evolved. Yet none of them appears to take cognisance of well-marked infringements of at least the spirit of these anti-advertising conventions. To mention merely one instance, the " Medical Directory " may be cited. In some impertinent quarters this useful compendium, which we willingly admit contains much interesting information, is regarded as the most absurd and injudicious kind of advertising, and when one compares it with the " Law List," for example, it is clear that there are very many objectionable points about it. No similar production, so far as we are aware, exists in any other country. It is a volume that is filled by the claims of self-assertion. Medical directories cater for the public, whether openly so or discreetly, does not very much matter. And the layman, we can safely assert, cannot discriminate between the records. He must accept what he reads, and when he sees that Dr. This has only London behind his name, while Dr. That puts himself down as a student of no less than seven Continental universities of repute, it is not to be wondered that he classes the former as a dunce and the latter as a genius. Probably Dr. That has only visited these foreign centres for a couple of weeks at the most—in very few cases, indeed, is it a fact that he can truthfully claim to be a student of any one of them in the same sense that he is a student of his own hospital. But how is the layman to know this? Again, the fortunate writer of a paper flourishes his achievement above or under the limited lines of his colleague, who has never appeared in the pages of a professional journal. Here again the layman cannot discriminate, and accepts without question. There are, fortunately, members of the profession so conscientiously alive to the spirit of their professional conventions that they shun the very shadow of self-advertisement; and one reflects on them with relish when considering the disadvantages of conventional restraints. It is a relish only slightly tempered by dissatisfaction when it is remembered that the mass is not so scrupulously conscientious, and that advertisement, in some form or other, is a sign of modern specialism and scientific activity, and that it is gradually asserting itself in the ranks of the medical profession.

